# Thriving after cancer: a summary of the special collection on the benefits of exercise and nutrition in cancer survivorship

**DOI:** 10.1093/jncics/pkag032

**Published:** 2026-04-27

**Authors:** Justin C Brown, Leila Tchelebi

**Affiliations:** AdventHealth, Orlando, FL, United States; Northwell, New Hyde Park, NY, United States

Cancer care is undergoing a fundamental shift. As more individuals live longer after a cancer diagnosis, a new challenge has emerged for the field of cancer control: how to optimize long-term health, functional independence, and quality of life for a rapidly growing population of cancer survivors.^1^ Simultaneously, the global burden of cancer continues to rise, driven in part by modifiable risk factors such as physical inactivity, obesity, and poor dietary patterns. Together, these trends have expanded the scope of cancer control beyond treatment alone, renewing emphasis on prevention, survivorship, and the broader determinants of health.^2^

Within this evolving landscape, lifestyle behaviors, particularly physical activity and nutrition, have emerged as powerful yet underutilized tools in oncology. A large body of epidemiological evidence links lifestyle factors to cancer risk, and an expanding literature suggests that these behaviors may also influence outcomes after diagnosis, including treatment tolerance, functional recovery, quality of life, and overall survival. Approximately 1 year ago, the Editors of *JNCI Cancer Spectrum* announced the creation of a special collection on the benefits of exercise and nutrition in cancer survivorship. The 13 publications featured in this special collection highlight how lifestyle science is increasingly informing strategies to improve treatment experiences and promote long-term health among survivors, revealing the flurry of research activity on this topic.

One of the most pressing issues in modern oncology is preserving physical function among cancer survivors. Survivorship is often accompanied by declines in mobility, strength, and overall physical capacity resulting from treatment toxicities, comorbid conditions, and accelerated physiologic aging. These impairments can have profound implications for independence and quality of life. In this context, Brown and Yang report exploratory findings from a multicenter randomized trial that older adults with a history of cancer face a substantially higher risk of developing major mobility disability compared with individuals without cancer, and participation in a structured physical activity intervention reduced the risk of mobility disability by more than 40% among survivors.^3^ These findings underscore the potential for exercise to function not merely as a supportive health behavior but as a powerful intervention capable of preserving functional independence.

Additional studies in this collection reinforce the functional benefits of physical activity interventions. Bates-Fraser and colleagues evaluated a year-long digital physical activity intervention among participants enrolled in the Cancer Prevention Study-3 cohort and observed improvements in walking cadence and lower-extremity functional performance among participants who engaged with the program.^4^ The results highlight the promise of technology-enabled approaches to deliver scalable interventions that can reach large survivor populations. In a randomized study comparing a moderate-intensity exercise program supported by either a low (5) or high (20) number of supervised sessions, Spence and colleagues found that both low- and high-supervision exercise improved fatigue, sleep, anxiety, depression, and physical function. Greater supervision appeared to confer additional improvements in upper-extremity function and pain outcomes.^5^ Together, these findings highlight the importance of identifying interventions that balance effectiveness with scalability.

Lifestyle interventions are also increasingly being evaluated during active cancer treatment. Emerging evidence suggests that carefully designed exercise and nutrition interventions can be both safe and feasible during treatment. In this collection, Tan and colleagues report that a telehealth-delivered lifestyle program integrating supervised exercise and dietary counseling during chemotherapy for breast cancer was associated with improvements in physical activity behavior.^6^ In another randomized trial, Puklin and colleagues compared a year-long lifestyle intervention initiated during chemotherapy for breast cancer with usual care and found that although declines in quality of life during treatment were not prevented, most outcomes returned to baseline levels within 1 year.^7^ Pahulu and colleagues conducted a systematic review of dietary interventions for prostate cancer survivorship, concluding that these interventions are safe and feasible but that additional clinical trials are needed.^8^ These findings illustrate both the potential and the limitations of lifestyle interventions during active therapy, suggesting that maintaining healthy behaviors during treatment may support longer-term recovery.

Safety concerns have historically limited the adoption of exercise interventions in oncology, particularly among breast cancer survivors at risk for lymphedema. For decades, clinicians advised patients to avoid upper-body exercise because of fears that it might worsen lymphatic swelling. The systematic review and meta-analysis by Plinsinga and colleagues provide strong evidence that such concerns may be largely unfounded.^9^ Across multiple studies, exercise was not associated with an increased risk of developing lymphedema and often improved symptoms such as pain and upper-extremity function. However, the authors highlight that many exercise trials lack detailed reporting of intervention dose, adherence, and progression. Improving the rigor and transparency of intervention reporting (eg, frequency, intensity, time, type, volume, and progression [FITT-VP]) will therefore be essential for advancing the field.

Lifestyle behaviors may influence clinical cancer outcomes through multiple biological pathways. Cao and colleagues examined the relationship between body mass index (BMI) and chemotherapy completion among patients with ovarian cancer and found that higher BMI was associated with higher relative dose intensity during chemotherapy.^10^ These findings challenge long-standing assumptions regarding treatment tolerance and highlight the importance of understanding body composition in cancer care. At the molecular level, Nakano and colleagues examined the association between dietary folate intake of 1-carbon nutrients and colorectal cancer risk by TP53 tumor status, suggesting that the effects of specific nutrients may differ by tumor molecular characteristics.^11^

The potential influence of lifestyle behaviors on cancer survival remains an area of active investigation. In a study of colorectal cancer patients, Smit and colleagues reported that increases in physical activity at specific time points after cancer diagnosis were associated with improved survival across multiple prognostic subgroups.^12^ In contrast, Peeri and colleagues’ analysis of pancreatic cancer survival overserved an association with prediabetes, but not life-course physical activity or BMI.^13^ These heterogeneous findings highlight the nuances of lifestyle epidemiology and underscore the need for additional translational epidemiologic research.

Understanding how exercise and nutrition influence tumor biology remains a key focus for cancer survivorship research. In a scoping review, Kang and colleagues examine the relationship between exercise intensity and immune function in cancer populations and report that vigorous-intensity exercise interventions were more frequently associated with improvements in immune parameters such as natural killer cell activity than lower-intensity exercise.^14^ Although no trials directly compared exercise intensities within the same study, these findings raise important questions regarding the FITT-VP characteristics of exercise required to produce meaningful biological effects. Bonhof and colleagues report the methods for the ongoing RADICES trial (the effect of exeRcise And Diet on quality of life in patients with Incurable Cancer of Esophagus and Stomach), which will expand our knowledge of the effects of exercise and nutrition in patients with incurable gastroesophageal cancer.^15^

The studies assembled in this Exercise and Nutrition special collection reflect the maturation of a field that is increasingly central to cancer control. Physical activity can preserve mobility and independence in survivors, lifestyle interventions can improve symptoms and quality of life during treatment, and emerging evidence suggests that exercise and nutrition may influence biological pathways relevant to tumor progression. Integrating lifestyle science into oncology research and clinical care will be essential for advancing a comprehensive vision of cancer control—a vision that extends beyond treating disease to promoting long-term health and well-being for patients and survivors. In an era defined by precision oncology, integrating exercise and nutrition into cancer care may represent 1 of the most powerful and accessible strategies for improving outcomes across the cancer continuum.

## Data Availability

No new data were generated or analyzed for this editorial.
